# No Significant Effect of Prefrontal tDCS on Working Memory Performance in Older Adults

**DOI:** 10.3389/fnagi.2015.00230

**Published:** 2015-12-14

**Authors:** Jonna Nilsson, Alexander V. Lebedev, Martin Lövdén

**Affiliations:** Aging Research Center, Karolinska Institutet and Stockholm UniversityStockholm, Sweden

**Keywords:** tDCS, working memory, plasticity, aging, N-back

## Abstract

Transcranial direct current stimulation (tDCS) has been put forward as a non-pharmacological alternative for alleviating cognitive decline in old age. Although results have shown some promise, little is known about the optimal stimulation parameters for modulation in the cognitive domain. In this study, the effects of tDCS over the dorsolateral prefrontal cortex (dlPFC) on working memory performance were investigated in thirty older adults. An N-back task assessed working memory before, during and after anodal tDCS at a current strength of 1 mA and 2 mA, in addition to sham stimulation. The study used a single-blind, cross-over design. The results revealed no significant effect of tDCS on accuracy or response times during or after stimulation, for any of the current strengths. These results suggest that a single session of tDCS over the dlPFC is unlikely to improve working memory, as assessed by an N-back task, in old age.

## Introduction

Aging is associated with cognitive decline, particularly in domains of working memory, executive function and episodic memory (Schaie, 1994). Whilst the negative consequences of poor cognition are well-known (Allaire and Marsiske, 2002; Salthouse, 2012), there is no universally accepted method for alleviating cognitive decline in old age (Zimerman and Hummel, 2010). Pharmacological approaches for preventing or delaying cognitive decline in old age have been unsatisfactory, urging an exploration of alternative strategies (Shafqat, 2008).

Transcranial direct current stimulation (tDCS) has been put forward as an attractive option for modulating perceptual, cognitive and behavioral functions, in the young and old, as well as in health and in disease (Nitsche et al., 2008; Zimerman and Hummel, 2010; Brunoni et al., 2012). The technique is non-invasive in nature and involves the induction of a weak constant current flow through the cerebral cortex via a positive electrode (anode) and a negative electrode (cathode) placed directly on the scalp. In the motor domain, anodal stimulation over the primary motor cortex increases motor evoked potentials (MEPs) induced by transcranial magnetic stimulation (TMS), indicating a modulatory effect on neuronal excitability in the stimulated area (Nitsche and Paulus, 2000). If the stimulation is applied for several minutes, these effects have been shown to outlast the stimulation itself by up to 90 min, evidencing a more long-lasting change in brain function (Nitsche and Paulus, 2001). The mechanisms underlying tDCS are poorly understood but the current understanding dictates that changes occurring during stimulation follows from an acute modulation of the resting transmembrane potential towards depolarization (under the anode) or hyperpolarization (under the cathode), making the neurons more or less likely to fire, respectively. In contrast, the changes observed after the stimulation are thought to be underpinned by long-term potentiation-like and long-term depression-like plastic processes (Nitsche et al., 2003; Horvath et al., 2015b).

While the effects of tDCS on MEPs are well established (Horvath et al., 2015a), effects on cognition have not been as consistent (Berryhill et al., 2014). In fact, a recent meta-analysis in younger adults failed to detect a reliable effect of a single session of tDCS on any of the included cognitive measures, independently of whether measures were considered separately or as part of broader cognitive domains (Chhatbar and Feng, 2015; Horvath et al., 2015a). Although the evidence base is much more limited for older adults, a recent meta-analysis that considered studies using tDCS as well as TMS revealed more promising results, with a moderate effect size (0.42) across all the included cognitive measures (Hsu et al., 2015). Interestingly, an even greater effect size (1.35) was revealed in a separate analysis that focused on patients with Alzheimer’s disease. It has therefore been suggested that the effects of tDCS may be dependent on the capacity of the individual, with potentially larger gains at the lower baseline levels commonly seen in old age and neurodegenerative disease (Hsu et al., 2015).

An important consideration relative to the motor domain is that there are several potential stimulation sites and behavioral outcomes for evaluating the effects of tDCS on cognition. The resulting heterogeneity between studies is reflected in the meta-analyses described above, which have included studies using a wide range of stimulation parameters (e.g., target site, stimulation duration) and even different stimulation techniques (e.g., tDCS, TMS). A more sensible analysis can be achieved by only including studies that target the same brain area and use the same outcome measure. In the working memory domain, the stimulation site has been relatively consistent with several studies implementing anodal stimulation over the left dorsolateral prefrontal cortex (dlPFC; Horvath et al., 2015a). Given the established involvement of this region in working memory, there is indeed a clear theoretical motivation for selecting this as a target site (Wager and Smith, 2003; Nee et al., 2007). Outcome measures have varied more, but the N-back task, which requires participants to respond when the stimulus shown is the same as N trials back, has featured frequently in tDCS studies that target working memory. In a meta-analysis in adults, anodal tDCS over the dlPFC was found to result in shorter reaction times in the N-back task (Brunoni and Vanderhasselt, 2014). Similarly, in one of the sub analyses presented by Horvath et al. (2015a), a trend towards a significant effect of anodal dlPFC stimulation on accuracy in the N-back task was detected (*p* = 0.065; Horvath et al., 2015a). Whilst not fully convincing, these findings indicate that consistency in regards to target site and outcome measure may increase the sensitivity of the analyses. Unfortunately, an analysis of equal specificity has not yet been conducted in old individuals and it therefore remains unknown whether tDCS over the dlPFC can improve working memory in old age.

Adding to target site and outcome measure, there are other stimulation parameters that are rarely given sufficient consideration. For example, not much is known about how current strength and stimulation duration influences the development of tDCS effects in the cognitive domain. In younger adults, individual studies have demonstrated greater cognitive gains following 2 mA compared to 1 mA (Iyer et al., 2005) but results have not been consistent (Hoy et al., 2013). In regards to stimulation duration, Ohn et al. (2008) found that the effects of anodal left dlPFC stimulation on N-back performance developed gradually during the 30 min long stimulation period, becoming reliable only at the end of stimulation. However, significant effects on N-back performance has also been demonstrated after only 10 min of stimulation over the left dlPFC (Fregni et al., 2005). Even less is known about the impact of such stimulation parameters in old individuals. Given the widespread changes in brain physiology and brain plasticity in old age, optimal tDCS parameters can reasonably be expected to differ compared to younger adults (Zimerman and Hummel, 2010; Fertonani et al., 2014).

The present study aimed to provide a detailed investigation of the effect of anodal tDCS over the left dlPFC on working memory performance in older individuals. To investigate how the effect develops over time, N-back performance was assessed before, three times during, 5 min after and 30 min after the 25 min long stimulation period. Furthermore, by implementing tDCS stimulation at both 1 mA and 2 mA, as well as sham stimulation, the study explored the importance of current strength. Based on previous meta-analytic results demonstrating a positive effect of brain stimulation on cognition in old age (Hsu et al., 2015), it was hypothesized that tDCS over the left dlPFC would improve working memory performance in old individuals. More robust effects were expected for higher current strengths, in the later phase of stimulation and after the stimulation has ended.

## Materials and Methods

### Subjects

Thirty older healthy adults (14 females) between 65 and 75 years participated. Average age was 69 ± 7 (*SD*) years. Participants had 14 ± 4 (*SD*) years of formal education. Data collection was conducted according to the Declaration of Helsinki. Ethical approval was obtained from Regionala Etikprövningsnämnden (Stockholm, 2014/2188-31/1). All subjects gave written informed consent in accordance with the Declaration of Helsinki.

### Experimental Design and Task

The study was designed as a single-blind, crossover, sham-controlled experiment. All participants received anodal tDCS over the left dlPFC at intensities of 1 mA and 2 mA as well as sham stimulation. The stimulation order was randomized and counterbalanced across participants. The interval between sessions was 48 h.

**Figure 1:**
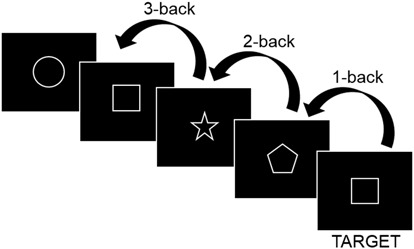
**Figural three-back task.** A target constitutes a shape that is the same as the shape presented three steps back in the series.

A figural three-back task assessed working memory performance (Figure 1). A three-back task was favored over a two-back tasks based on ceiling performance for some participants in the two-back tasks in an informal pre-testing session. For each task run, which lasted for 1 min, participants were presented with a pseudorandom series of 22 shapes drawn from a set of six shapes (circle, square, hourglass, triangle, star, pentagon). Each shape was presented for 2250 ms with an interstimulus interval of 250 ms. For each presented shape, participants were required to respond only when the shape constituted a target, i.e., when the shape was same as the shape presented three steps before (Figure 1). There were six targets per task run. Four 1 min task runs with 20 s rest between each run constituted one N-back assessment, resulting in a total duration of 5 min per assessment.

**Figure 2:**
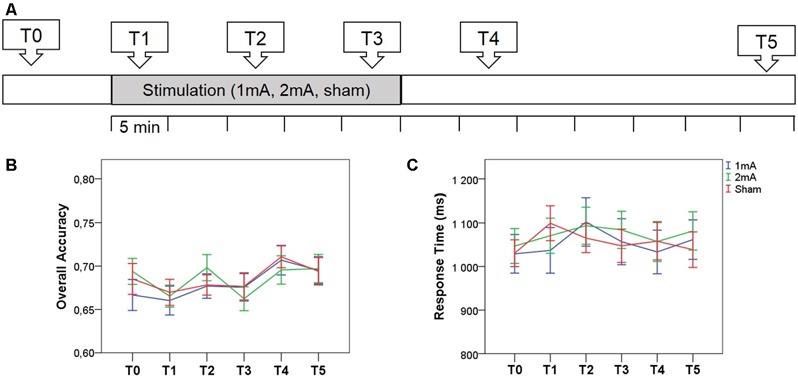
**(A)** Experimental design. Three-back performance was assessed before (T0), during (T1−T3) and after (T4−T5) anodal tDCS over the left dlPFC. Stimulation conditions were randomized for each participant and the order was counterbalanced. **(B,C)** The effect of stimulation condition on overall accuracy and response time at the six assessment time points. Error bars represent standard error of the mean.

N-back performance was assessed six times per session: before tDCS, during tDCS at 0–5 min (T1), at 10–15 min (T2) and at 20–25 min (T3), and after tDCS, at 5–10 min (T4) and 30–35 min (T5; Figure 2A). Participants were familiarized with the task for 30 min prior to the experiment.

### tDCS

Direct current was delivered using the DC-STIMULATOR PLUS (neuroConn GmbH, Ilmenau, Germany) and was transferred by two saline-soaked surface electrodes. The anode (7 × 5 cm) was centered over F3 according to the 10–20 international system for EEG placement. Models of current flow intensity and distribution have provided support for F3 for targeting left dlPFC (Seibt et al., 2015). The cathode (10 × 10 cm) was placed over the contralateral supraorbital area. In all sessions, the current was initially increased in a ramp-like fashion over 8 s. In the active stimulation conditions, a constant current was delivered for 25 min at an intensity of 1 mA or 2 mA. The stimulation duration was set to 25 min to allow the completion of three N-back assessments during stimulation whilst also allowing five minutes rest in between each assessment. In the sham condition, the current was terminated after 40 s. After each session, participants guessed which stimulation they thought they had received.

### Statistical Analyses

The primary outcome was overall accuracy and response time on accurate trials during/after tDCS. Overall accuracy was the averaged sum of true positives (number of hits/total targets) and true negatives (number of correct rejections/total foils) with a nominal chance level at 0.5. The effect of stimulation on N-back performance was evaluated in repeated-measures analyses of variance with time (T0–T5) and stimulation (1 mA, 2 mA, sham) as within-subject factors. Wilcoxon signed-ranks tests were used to assess blinding by comparing the accuracy of participants’ guesses to chance levels (0.3). Analyses were performed using IBM SPSS Statistics 22 software (Chicago, IL, USA). The alpha level was *p* < 0.05.

## Results

Baseline performance differed significantly from chance at 0.68 ± 0.07, *t*_(29)_ = 13.459, *p* < 0.001. Accuracy and response times at baseline did not differ between stimulation conditions (all *p*s > 0.09). For accuracy, there was no main effect of stimulation, *F*_(2,58)_ = 0.240, *p* = 0.79, and no interaction between time and stimulation, *F*_(10,290)_ = 0.921, *p* = 0.51 (Figure 2B, Supplementary Table). The main effect of time was significant, *F*_(5,145)_ = 6.876, *p* < 0.001, likely reflective of a learning effect. For response times, there was no main effect of stimulation, *F*_(2,58)_ = 0.423, *p* = 0.66, or time, *F*_(5,145)_ = 1.527, *p* = 0.19, and no interaction between stimulation and time, *F*_(10,290)_ = 0.914, *p* = 0.52 (Figure 2C, Supplementary Table). Paired *t*-tests revealed no significant group differences at any of the time points (all *p*s> 0.07). Considering change in performance between baseline and the last offline assessment, there was no effect of stimulation condition on accuracy, *F*_(2,58)_ = 0.763, *p* = 0.47, or response times, *F*_(2,58)_ = 0.264, *p* = 0.769. There were also no relationships between change in any of the stimulation conditions and average baseline performance, age or education (all *p*s > 0.08, no correction for multiple tests).

In regards to blinding, participants’ guesses did not differ from chance after having experienced one and two stimulation conditions, both *Z* = −0.907, *p* = 0.36, but after having experienced all conditions guesses exceeded chance levels (*Mdn* = 1.00), *Z* = −3.555, *p* < 0.001.

## Discussion

The results revealed no significant effect of anodal tDCS over the left dlPFC on working memory performance in older adults. Relative to sham, tDCS at a current strength of 1 mA or 2 mA did not modulate performance at any point during stimulation or after stimulation. The hypothesis that working memory can be improved with a single session of anodal tDCS over the left dlPFC in older adults could therefore not be supported.

The lack of a robust cognitive effect after a single session of tDCS over the left dlPFC can be considered consistent with the results in younger individuals (Horvath et al., 2015a). Although the results of brain stimulation techniques on cognition has been more promising in old individuals (Hsu et al., 2015), findings relating directly to the effect of anodal tDCS over the dlPFC on N-back performance has to our knowledge been limited to a single study (Berryhill and Jones, 2012). Unfortunately, the methodology used by Berryhill and Jones (2012) differs both in terms of the specifics of the stimulation protocol and the N-back task, which makes a direct comparison difficult. A possibility therefore is that the particular stimulation protocol used in the present study may not have been optimal or that working memory itself, as assessed by a figural three-back task, is not amenable to tDCS modulation in old age.

Another possibility is that the effect developed over a longer time period than that incorporated in the study design. In support of this, evidence has started to emerge that older individuals may exhibit a delayed response to tDCS. In the motor domain, the maximum response to tDCS has been shown to occur 30 min later in old individuals than for young individuals (Fujiyama et al., 2014). Similarly, in a cognitive training study with repeated stimulation sessions in older adults, benefits of tDCS were not detected until one month after the last stimulation session (Jones et al., 2015). Furthermore, the meta-analysis that investigated the effect of TMS and tDCS in older adults found evidence of a more reliable effect in studies that assessed cognition after the stimulation had ended (Hsu et al., 2015). It is therefore possible that the maximum response to tDCS occurred after the last N-back assessment at 30 min post-stimulation in the present study.

A single session of tDCS may not be sufficient to produce robust cognitive change. Considering the proposed involvement of NMDA receptors and long-term potentiation and depression in the enduring effects of tDCS (Nitsche et al., 2003), repeated stimulation sessions may be required (Hsu et al., 2015). In particular, combining repeated tDCS with cognitive training has emerged as a promising approach in young and well as in older adults (Martin et al., 2013; Park et al., 2014; Richmond et al., 2014; Jones et al., 2015). If tDCS can indeed be understood as a facilitator of synaptic plasticity, an ongoing learning process may be necessary to achieve robust and long-lasting cognitive improvements.

In the context of the possibilities discussed above, the cross-over design employed in the present study is an important limitation (Brunoni and Vanderhasselt, 2014). First, if tDCS effects continue to develop post-stimulation, a wash-out period of 48 h may have been insufficient. Second, in the case of ongoing learning across study visits, the order of the stimulation protocols (1 mA, 2 mA, sham) may have been influenced the effects on working memory. Whilst the order was counterbalanced across subjects and therefore controlled for in the design, interactions between tDCS effects and the learning process may have introduced sufficient noise to prevent an effect from being detected. Related to the limitations of a cross-over design is the challenge to maintain blinding. At the end of the present study, participants were able to guess which stimulation they had received at which visit at a higher accuracy than would be expected by chance. Going forward, a between-subject design should therefore be favored.

Like the majority of previous tDCS studies, the present study was also limited in terms of statistical power. *Post hoc* power calculations indicate that our power to detect a significant pairwise difference was limited even for large effect sizes (*effect size* = 0.5 SD, α = 0.05 and *n* = 30 gives 1-β = 0.75, G × Power 3, (Faul et al., 2007). Meta-analyses on tDCS on cognition have suggested effect sizes in the small to medium range (Brunoni and Vanderhasselt, 2014; Horvath et al., 2015b; Hsu et al., 2015), which therefore may have gone undetected in our study. However, considering an average sample size of 21 in cognitive investigations of tDCS over dlPFC (Tremblay et al., 2014), it should be emphasized that limited power represents a general challenge in the field. It is therefore critical that results from individual studies are reported, independent of outcome, to enable meta-analyses with good statistical power.

In conclusion, the present study found no evidence in support of the hypothesis that a single session of anodal tDCS over the left dlPFC improves working memory performance in old age. Future investigations will be necessary to explore whether tDCS, repeated and in combination with learning, could be a more promising approach for alleviating cognitive decline in old age.

## Author Contributions

All authors made substantial contributions to the conception or design of the work, acquisition, analysis and interpretation of the data. The corresponding author took the lead for writing the manuscript but all authors made important revisions and provided their final approval.

## Funding

This research was funded by the European Research Council (Consolidator Grant REBOOT 617280).

## Conflict of Interest Statement

The authors declare that the research was conducted in the absence of any commercial or financial relationships that could be construed as a potential conflict of interest.
