# Supplementary material for: No Significant Effect of Prefrontal tDCS on Working Memory Performance in Older Adults
Source: Front Aging Neurosci. 2015 Dec 14;7:230. doi: 10.3389/fnagi.2015.00230 (PMC4677281; doi:10.3389/fnagi.2015.00230)

Supplementary Material

**No significant effect of prefrontal tDCS on working memory performance in older adults**

**Jonna Nilsson *, Alexander Lebedev, Martin Lövdén**

*** Correspondence:** Jonna Nilsson, [jonna.nilsson@ki.se](mailto:jonna.nilsson@ki.se)

# Supplementary Table 1: Means and standard deviations for the three stimulation conditions (1mA, 2mA and sham) at the six different time points (T0-T5).


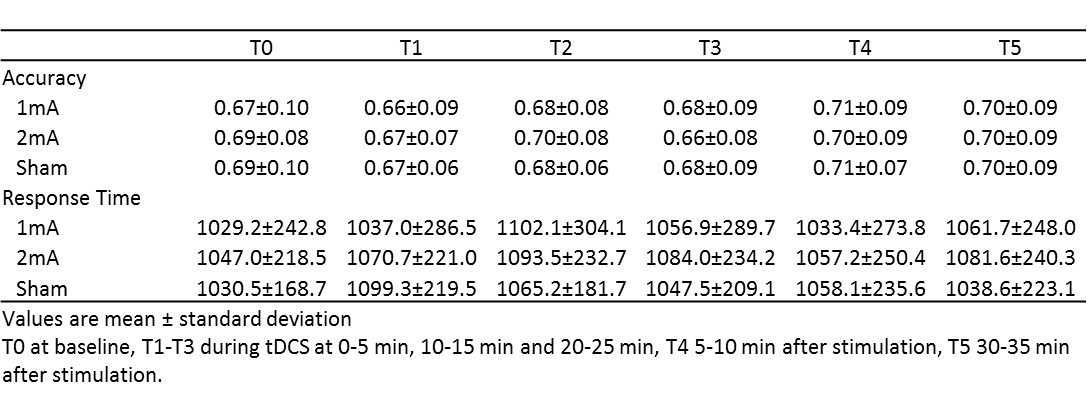

Supplement: Supplementary file 1 [file Data_Sheet_1.docx]
